# Current Anticoagulation Statuses among Older Chinese People with Nonvalvular Atrial Fibrillation

**DOI:** 10.31083/j.rcm2503079

**Published:** 2024-02-29

**Authors:** Junrong Jiang, Yihan Weng, Jun Huang, Hai Deng, Hongtao Liao, Xianhong Fang, Xianzhang Zhan, Shulin Wu, Yumei Xue

**Affiliations:** ^1^Guangdong Cardiovascular Institute, Guangdong Provincial People’s Hospital, Guangdong Academy of Medical Sciences, 511436 Guangzhou, Guangdong, China; ^2^Guangdong Provincial Key Laboratory of Clinical Pharmacology, Guangdong Provincial People's Hospital, Guangdong Academy of Medical Sciences, 511436 Guangzhou, Guangdong, China; ^3^Department of Geriatrics, Guangdong Provincial People’s Hospital, Institute of Geriatrics, Guangdong Academy of Medical Sciences, 511436 Guangzhou, Guangdong, China

**Keywords:** atrial fibrillation, anticoagulation, older people, China

## Abstract

**Background:**

The reported anticoagulation rate may be overestimated among Chinese patients with atrial fibrillation (AF). Therefore, we aimed to understand the current status and time trends of anticoagulation among older people in the Chinese community.

**Methods:**

Data were obtained from the physical examination program for the elderly (aged ≥65 years) in Guangzhou. During 2017–2020, a total of 31,829, 58,573, 55,483, and 54,845 older people underwent annual physical examinations, respectively, where their general information, AF-related medical history, and use of oral anticoagulants (OACs) were collected for analysis.

**Results:**

From 2017 to 2020, the estimated annual prevalence of older people with nonvalvular atrial fibrillation (NVAF) in Guangzhou was 0.99%, 0.92%, 1.05%, and 1.14%, respectively. In patients with high stroke risk (CHA2DS2-VASc score ≥2 for males or ≥3 for females), the annual anticoagulation rates were 2.83%, 2.05%, 5.29%, and 5.82%, respectively. The proportion of NVAF patients prescribed non-vitamin K antagonist oral anticoagulants (NOACs) increased gradually over the same period (*p* = 0.004). Males (odds ratios (OR), 1.797; 95% confidence interval (CI), 1.169–2.763; *p* = 0.008), ages over 75 (OR, 1.858; 95% CI, 1.212–2.849; *p* = 0.005), low education levels (OR, 1.737; 95% CI, 1.132–2.665; *p* = 0.011), and lacking the ability for self-care (OR, 4.432; 95% CI, 1.067–18.418; *p* = 0.041) were less likely to receive OAC therapy.

**Conclusions:**

The low anticoagulation rate of older people with NVAF in the Chinese community has not significantly improved in recent years, with only 5.82% of patients with high stroke risk being prescribed OACs. Therefore, it is necessary to establish an appropriate mode of anticoagulant management to improve the current situation.

## 1. Introduction

Atrial fibrillation (AF) is the most common sustained arrhythmia with a global prevalence of approximately 2%–4% [1]. Prevalence is expected to increase even further owing to the increased longevity of the general population, ineffective control of risk factors, and improved diagnostic capabilities [2, 3]. AF can significantly increase the risk of stroke [4]. Compared to stroke without AF, AF-related stroke is more fatal and disabling [5]. Oral anticoagulants (OACs) are recommended to reduce the thromboembolic risk in AF patients with moderate to high stroke risks [6]. Previous studies suggested that more than 50% of AF patients with high risks of stroke were treated with OACs in developed countries [7, 8, 9], while in China, only 36.5% were reported to be treated with OACs [10].

However, since most of the available data are from medical institutions with high-quality health care, the current status of OACs in China may even be overestimated. At present, the medical level in China lags behind that of developed Western countries, and the distribution of medical resources is uneven. Owing to the large population and limited quality of medical resources, the task of diagnosing and treating common diseases in China mainly depends on primary community health institutions. Therefore, data from primary community health institutions may more accurately reflect the current anticoagulation status. To this end, we sought to increase the understanding of anticoagulation among older people with nonvalvular atrial fibrillation (NVAF) in Chinese communities by analyzing the physical examination data from primary community health institutions.

## 2. Methods

### 2.1 Data Source

Data were obtained from the physical examination program for older people in Guangzhou, southern China. The project was funded by the local government and conducted at primary community health institutions annually. People over the age of 65 voluntarily participated. This project aimed to understand the health profile of older people and assist in the development of efficient public health strategies for geriatric chronic diseases. Information, such as sociodemographic characteristics, medical history, laboratory tests including blood routine, liver and kidney function, electrolytes, lipids, electrocardiogram (ECG), and chest radiograph, was collected from participants. The collection and aggregation of data are the responsibility of the local Center for Disease Control and Prevention (CDC).

### 2.2 Study Population

With the authorization of the local government and CDC, this study obtained physical examination data from older people between 2017 and 2020 in the Yuexiu District of Guangzhou. Approved by the ethics committee of Guangdong Provincial People’s Hospital, this study was conducted in accordance with the Declaration of Helsinki and Good Clinical Practice Guidelines. Since the information was retrospectively collected, the requirement for informed consent was waived. The permanent population of Yuexiu District is around 1.15 million, with 13% of the population aged 65 and above. There are 18 primary community health institutions in the area. During 2017–2020, a total of 31,829, 58,573, 55,483, and 54,845 older people underwent annual physical examinations in the Yuexiu District, respectively. General information (e.g., sex, age, body mass index (BMI)), AF-related medical history (e.g., hypertension, diabetes mellitus, coronary artery disease, heart failure, stroke, vascular disease), and use of OACs were collected for analysis.

### 2.3 Statistical Analysis

Baseline characteristics were reported as proportions for categorical variables and mean ± standard deviation (SD) for continuous variables. Categorical variables were compared using the χ^2^ test and log-rank test, while continuous variables were compared using the independent *t* test. Independent risk factors associated with OACs were determined by stepwise logistic regression analysis. The odds ratios (ORs) and corresponding 95% confidence intervals (95% CIs) were calculated to assess the associations. SAS software version 9.4 (SAS Institute Inc.; Cary, NC, USA) was used for the statistical analyses. All statistical tests were two-sided, and *p*-values less than 0.05 were considered statistically significant.

## 3. Results

### 3.1 Annual Patient Characteristics

**Fig. 1.**
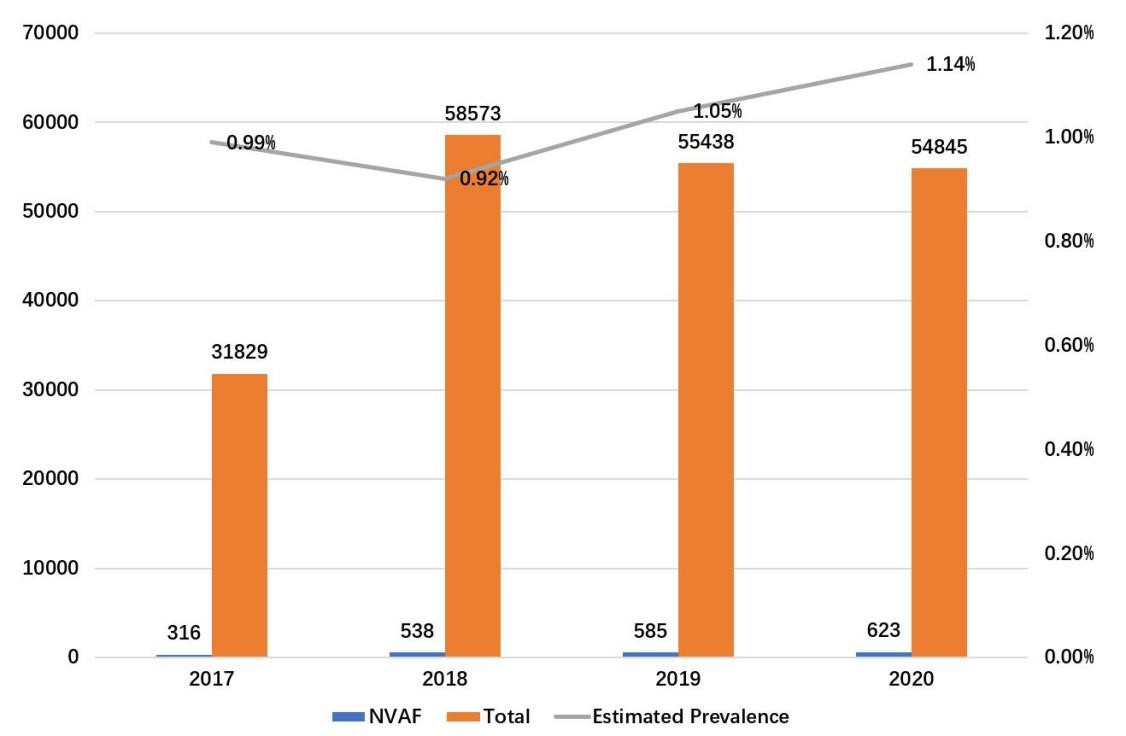
**The annual prevalence of NVAF**. NVAF, nonvalvular atrial fibrillation.

Annual patient characteristics for each year between 2017 and 2020 are summarized in Table 1. From 2017 to 2020, the estimated annual prevalence of older people with NVAF in Guangzhou was 0.99%, 0.92%, 1.05%, and 1.14%, respectively (Fig. 1). The proportion in males gradually increased, while the proportion of older people over 75 years old gradually decreased (*p* < 0.05). During this period, except for heart failure (*p* = 0.007), no significant difference was observed in the prevalence of comorbidities, including hypertension, diabetes, stroke, coronary heart disease, and peripheral vascular disease. Similarly, there was no significant difference in the proportion of individuals with high stroke risk (CHA2DS2-VASc score ≥2 for males or ≥3 for females).

**Table 1.** **Annual comorbidities in all patients with NVAF**.

	Year				*p*-value
	2017 (n = 316)	2018 (n = 538)	2019 (n = 585)	2020 (n = 623)	
Age (≥75 years)	192 (60.8)	334 (62.1)	326 (55.7)	337 (54.1)	0.021
Female	170 (53.8)	265 (49.3)	270 (46.2)	274 (44.0)	0.027
Heart failure	10 (3.2)	31 (5.8)	47 (8.0)	27 (4.3)	0.007
Hypertension	200 (63.3)	356 (66.2)	402 (68.7)	431 (69.2)	0.245
Diabetes	76 (24.1)	125 (23.2)	157 (26.8)	149 (23.9)	0.507
Stroke/TIA	36 (11.4)	66 (12.3)	89 (15.2)	76 (12.2)	0.276
Coronary heart disease	117 (37.0)	208 (38.7)	214 (36.6)	201 (32.3)	0.130
Peripheral vascular disease	7 (2.2)	10 (1.9)	8 (1.4)	9 (1.4)	0.749
CHA2DS2-VASc score ≥2 (males) or ≥3 (females)	283 (89.6)	488 (90.7)	529 (90.4)	550 (88.3)	0.515

TIA, transient ischemic attack; NVAF, nonvalvular atrial fibrillation.

### 3.2 Anticoagulation Rate and Selection of Oral Anticoagulants

Changes in anticoagulation use between 2017 and 2020 are summarized in Table 2. Regardless of the patients’ CHA2DS2-VASc scores, the annual anticoagulation rates among older people with NVAF were 3.16%, 3.16%, 5.64%, and 5.62%, respectively. No significant difference was shown in the overall anticoagulation rate. In patients with high stroke risk (CHA2DS2-VASc score ≥2 for males or ≥3 for females), the annual anticoagulation rates were 2.83%, 2.05%, 5.29%, and 5.82%, respectively. The use rate of OACs among patients with high-risk NVAF seemed to increase during the study period (*p* = 0.007). The proportion of NVAF patients prescribed non-vitamin K antagonist oral anticoagulants (NOACs) increased gradually over the same period (*p* = 0.004). Notably, approximately 8–11% of patients were prescribed antiplatelet drugs rather than OACs during the study period.

**Table 2.** **Anticoagulation rates and selection of oral anticoagulants**.

		Year				*p*-value
		2017 (n = 316)	2018 (n = 538)	2019 (n = 585)	2020 (n = 623)	
Anticoagulation rate (%)		3.16 (10/316)	3.16 (17/538)	5.64 (33/585)	5.62 (35/623)	0.074
Anticoagulation rate (%) in CHA2DS2-VASc ≥2 (male) or ≥3 (female)		2.83 (8/283)	2.05 (10/488)	5.29 (28/529)	5.82 (32/550)	0.007
OACs						0.004
	*Warfarin*	8	15	21	14	
	*NOACs*	2	2	12	21	
Antiplatelet drugs		25 (7.9)	52 (9.7)	70 (12.0)	68 (10.9)	——

OACs, oral anticoagulants; NOACs, non-vitamin K antagonist oral anticoagulants.

### 3.3 Factors Associated with Oral Anticoagulant Use

According to current guidelines, OACs should be considered for NVAF patients with CHA2DS2-VASc scores = 1 (male) or = 2 (female) [6]. Factors associated with OAC use were determined by stepwise multiple logistic regression analysis. Males (OR, 0.556; 95% CI, 0.362–0.855; *p* = 0.008), ages over 75 (OR, 0.538; 95% CI, 0.351–0.825; *p* = 0.005), low education levels (OR, 0.576; 95% CI, 0.375–0.883; *p* = 0.011), and lacking the ability for self-care (OR, 0.226; 95% CI, 0.054–0.937; *p* = 0.041) were less likely to receive OAC therapy (Table 3).

**Table 3.** **Factors associated with OACs by stepwise multiple logistic regression analysis**.

	No anticoagulation (n = 1967)	Anticoagulation (n = 95)	*p*-value	OR (95% CI)
Male	1040 (52.9)	43 (45.3)	0.008	0.556 (0.362–0.855)
Married	1567 (79.7)	85 (89.5)	0.217	——
Age (≥75 years)	1150 (58.5)	39 (41.1)	0.005	0.538 (0.351–0.825)
BMI	24.2 ± 3.5	23.8 ± 3.1	0.052	——
CHA2DS2-VASc ≥2 (male) or ≥3 (female)	1772 (90.1)	78 (82.1)	0.237	——
Alcohol	1785 (90.8)	82 (86.3)	0.190	——
Smoking	261 (13.3)	11 (11.6)	0.468	——
Exercise	1293 (65.7)	64 (67.4)	0.326	——
Education (under high school)	1276 (64.9)	50 (52.6)	0.011	0.576 (0.375–0.883)
Without medical insurance	48 (2.4)	3 (3.2)	0.616	——
Lacking self-care ability	208 (10.6)	2 (2.1)	0.041	0.226 (0.054–0.937)

BMI, body mass index; OACs, oral anticoagulants; OR, odds ratio.

## 4. Discussion

The present study is the first to examine trends in NVAF prevalence and OAC therapy for older people in China using data obtained from primary community health institutions. We found that the prevalence of NVAF in Guangzhou was around 1%, which was lower than previously reported [1]. According to the results of a previous epidemiological study in Guangzhou, the prevalence of AF in people over 60 was over 1.65%, while the prevalence in people aged 80 years and above was 5.0% [11]. Since there is a higher risk of significant mitral calcification or degenerative valve disease in older populations, it may limit the population from being diagnosed with NVAF [12]. Although the use rate of OACs among patients with high-risk NVAF (CHA2DS2-VASc score ≥2 for males or ≥3 for females) seemed to increase during the study period, the anticoagulation rate was still low. Only 5.82% of patients were prescribed OACs in 2020. Due to the great efforts made for stroke prevention, treatment with OACs in patients with NVAF seems to be improving in China [13]. A previous study showed that in-hospital OAC use in 2006 was 11% among patients with NVAF [14], while 36.5% of patients with CHA2DS2-VASc scores ≥2 received OACs according to data from a 2016 Chinese Atrial Fibrillation Registry Study [10]. However, most of the data was obtained from tertiary hospitals. There may be a large variation of OAC treatment between tertiary and non-tertiary hospitals [10]. Due to the outbreak of COVID-19 in late 2019, people were less likely to participate in physical examinations or attend primary community health institutions for follow-ups, which may partly explain the low anticoagulation rate. Moreover, due to the increased risk of death among older people caused by COVID-19, this study found that the proportion of NVAF patients aged over 75 years decreased annually.

Several studies suggested that the anticoagulation rate in the Chinese community was only 2.2%–7.8% from 2010 to 2016 [15, 16, 17], which was much lower than reported in the US and Europe [18]. The present study found that the anticoagulation rate in the community was only 5.8%, according to data from Guangzhou during 2017–2020. Since there was no obvious improvement in the low anticoagulation rate during this period, the status of OAC use in the Chinese community remains a concern. According to the results of a previous survey, which we conducted in the community of Guangzhou, multiple factors may contribute to the insufficient anticoagulation treatment observed in the community. First, standardized training in AF management is lacking among physicians in the community. Second, there is no NOAC and coagulation monitoring equipment in primary community health institutions. Additionally, concerns over bleeding impair the willingness of physicians and patients to use OACs. Finally, antiplatelet agents have been inappropriately used in China to prevent stroke among patients with AF [19]. In the present study, 8–11% of patients were still prescribed antiplatelet agents for stroke prevention. However, the use of antiplatelet agents is neither safe nor effective for preventing stroke in patients with AF [20].

In China, a large number of patients in the community with AF remain exposed to the risk of stroke because of a lack of anticoagulants. Therefore, it is necessary to establish an appropriate mode of anticoagulant management for patients in the community with AF. Multiple effective modes of anticoagulant management have been explored in the community, similar to those in the US and Europe [21, 22, 23, 24]. Among them, National programs promoted by the UK and Canada seem to be effective modes that can increase the anticoagulation rate and improve quality of life [25, 26]. Notably, stepwise multiple logistic regression analysis suggested that being male, advanced in age (≥75 years), low education level, and lacking the ability for self-care were associated with the underuse of OACs since these factors may be detrimental to disease awareness and adherence.

## 5. Limitations

There were several limitations in this study. First of all, since the data were collected from Guangzhou, an economic center in China, the results are not fully representative of the whole country, especially in less-developed regions. Therefore, it may lack generalizability outside of China. Second, this study only analyzed the current status and time trends of oral anticoagulation use in older people, those aged over 65 years old. Finally, this study was not able to analyze the duration of anticoagulation use and changes in OAC selection for each patient because of limitations in the data.

## 6. Conclusions

In conclusion, the low anticoagulation rate in older people with NVAF in the Chinese community has not significantly improved in recent years, with only 5.82% of patients with high stroke risk (CHA2DS2-VASc score ≥2 for males or ≥3 for females) being prescribed OACs. Compared to tertiary hospitals, the status of anticoagulation use in the community remains worrying. Thus, it is necessary to establish an appropriate mode of anticoagulant management to improve the current situation.

## Data Availability

The data that support the findings of this study are available from Health Bureau and CDC of Yuexiu District but restrictions apply to the availability of these data, which were used under license for the current study, and so are not publicly available. Data are however available from the authors upon reasonable request and with permission of Health Bureau and CDC of Yuexiu District. If necessary, please contact Junrong Jiang (571023647@qq.com).
